# Reducing antimicrobial resistance burden from livestock production by targeting pig gut health

**DOI:** 10.3389/fvets.2026.1854400

**Published:** 2026-06-29

**Authors:** Charlotte Lauridsen, Hauke Smidt, Merete Fredholm, Maria L. Marco, Manimozhiyan Arumugam

**Affiliations:** 1 Department of Animal and Veterinary Sciences, Aarhus University, Foulum, Denmark; 2 Laboratory of Microbiology, Wageningen University and Research, Wageningen, Netherlands; 3 Department of Veterinary and Animal Sciences, University of Copenhagen, Frederiksberg, Denmark; 4 Department of Food Science and Technology, University of California Davis, Davis, CA, United States; 5 Novo Nordisk Foundation Center for Basic Metabolic Research, University of Copenhagen, Copenhagen, Denmark

**Keywords:** antimicrobial resistance, feed, genomics, microbiome, phenomics, pigs

## Abstract

Antimicrobial resistant bacterial infections are increasing at an alarming rate and are expected to result in a catastrophic humanitarian health crisis as more deaths due to antimicrobial resistance (AMR) than cancer is foreseen by year 2050. Our mission is to perform research that can benefit people and societies in the fight against AMR. The animal food production system has been a primary user of antibiotics for growth promotion, and although this was banned in the EU in 2006, conventional pig production remains to be a major consumer of antibiotics for treatment of infections. This paper presents the perspective of our project ‘Preventing Infection in the Gut of developing Piglets -and thus Antimicrobial Resistance - by disentangling the interface of diet, the host and the Gastrointestinal Microbiome’ (PIG-PARADIGM, https://projects.au.dk/pig-paradigm). The vision of PIG-PARADIGM is to reduce the overall need for antimicrobial treatments and mitigate the spread of AMR by delivering fundamental knowledge on (i) what defines healthy and robust intestinal function in pigs; (ii) what determines the host and microbial mechanisms leading to post weaning diarrhea and subsequent antibiotic use; (iii) how the intestinal microbiome and nutrition can be modulated to prevent the need for antibiotic use by promoting resilience to early life stress and intestinal infections; and (iv) how AMR can be minimized through increased intestinal resilience. Outcome is the knowledge on enhancing pig intestinal resilience and reducing diarrheal disease incidence, whereby the need for antibiotic use will be significantly reduced as will the risk for AMR pathogen emergence and spread.

## 1 Introduction

Antimicrobial resistance (AMR) is a natural phenomenon that is exacerbated by the misuse and overuse of antimicrobials threatening human and animal health and welfare (1). Animal agriculture has been a primary user of antibiotics for growth promotion, and although this was banned in the EU in 2006, conventional pork production still requires heavy antibiotic use (2). Pigs are especially exposed to antibiotic treatments during the weaning transition when diarrhea is frequent, the so-called post weaning diarrhea (PWD). To replace the use of antibiotics for the treatment of diarrhea in weaned pigs, a common practice was the high-dose zinc oxide (ZnO) in piglet feed (Supplementary Figure S1). However, in June 2022 EU implemented a ban on this therapeutic use forcing producers to find alternatives to control PWD and reduce antibiotic reliance. While nutritional zinc levels (around 150 ppm) are still allowed, the elimination of the high pharmacological doses have led to an increased use of antimicrobials as a temporary consequence (3).

Efforts to stop or reduce antibiotic use in livestock production are now underway or already implemented in some countries supported by, e.g., the International Centre for Antimicrobial Resistance Solutions.^1^ However, despite these changes, antibiotics remain crucial in the treatment of infectious diseases in livestock, whereby spreading of AMR remains a pervasive threat for human and animal health.

During the weaning transition of the pigs, the intestinal microbiome is highly affected by dietary changes, i.e., the change from suckling to solid feed, and by other external factors such as mixing of pigs, changing of environment and antibiotic use. Populated by trillions of microorganisms, including bacteria, archaea, fungi and other micro-eukaryotes, and viruses/phages, the intestine is not only vulnerable to disruption by antibiotics, but because of its high-density, the intestinal microbiome is also an important source of AMR genes spreading among bacterial species, including animal and human pathogens (4). Diarrhea and antibiotic use are persistent today because many unanswered questions remain about the animal and human intestinal microbiome including: “What constitutes a healthy microbiome?”, “What defines the cooperative relationship between the intestinal microbiome and host?”, and “How can diet affect this relationship?” Despite these knowledge gaps, it is generally accepted that there is a relationship between the intestinal microbiome and host physiology and pathology (5, 6). Diet composition is also an important modulator of the intestinal microbiome (7, 8) and can modulate host immunity (9) and intestinal health.

The scope of our innovative project, as outlined in this paper, is to understand the root causes of PWD in pigs; examine the relationships between intestinal health, antibiotic use, and AMR development in the gut microbiome; identify biomarkers for robust and resilient pig health; and establish dietary measures that sustain and repair intestinal health, preventing the need for antibiotic treatment. By increasing pig robustness, and at the same time being able to predict deviations from it early on, we expect that the need for antimicrobials will be significantly diminished, as will eventually AMR. Although ambitious, this goal could enable antibiotic-free pig production, except for occasional veterinary interventions, within the next 10 to 15 years. Realizing the complexity of the research topic and the multidisciplinary approaches needed to develop the fundamental knowledge on what defines a healthy and robust intestinal function in pig, we have arranged research-based concepts into four complementary, yet strongly intertwined and interacting pillars consisting of three biological pillars entitled ‘Host’, ‘Microbiome’, and ‘Nutrition’ and a fourth pillar entitled ‘Data integration’ to harmonize and integrate the multi-omics host-microbiome-nutrition data generated in the three other pillars (Supplementary Figure S1). With this unique framework it will be possible to holistically model the host-microbiome holobiont in the developing pig and to obtain a knowledge base that enables (microbiome-) targeted dietary interventions that improve and/or support intestinal and systemic development and health by reducing the risk of infectious diarrhea during the post weaning period.

## 2 The host pillar

The Host Pillar involves a thorough clinical and pathological characterization of intestinal health in a cohort of 2,500 pigs. The motivation for the studies relies on the fact that, despite standardized procedures in management, feeding, climate control and disease prevention, significant variation exists in intestinal health and performance among individual pigs [e.g., Weber et al. (27)]. Factors such as large litter size, low parity sows, low birthweight, low colostrum uptake, and early weaning may partially explain these health differences. However, it is evident that pigs also have varying levels of robustness due to host genetics, which affect immune function, metabolism and other physiological parameters critical to systemic health [e.g., Cirera et al. (10), Frederiksen et al. (11), Rowland et al. (12), and Zhang et al. (13)]. Additionally, the intestinal microbiome significantly influences disease risk (14), while host genetics, in turn, affect intestinal microbiome composition (15). Therefore, an important goal within this pillar is to clarify the intricate interactions between the intestinal microbiome and host factors, as well as to determine their individual and combined effects on intestinal health.

**Figure 1:**
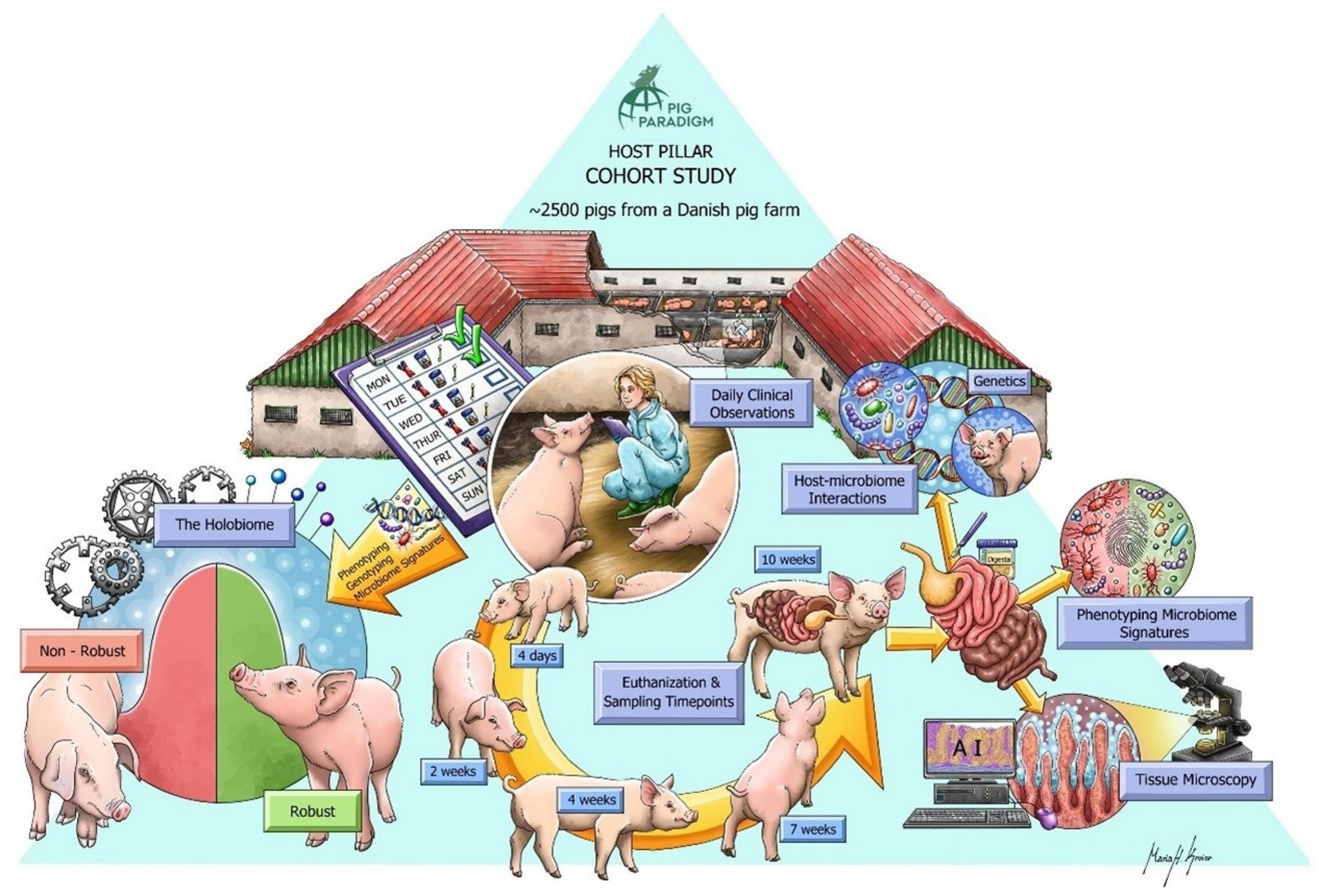
The Host Pillar in PIG-PARADIGM involves a thorough clinical and pathological characterization of intestinal health in a cohort of 2,500 pigs. More information of the project can be obtained from: https://projects.au.dk/pig-paradigm. (Figure created by Maria Kroier).

The overall structure of the pillar is divided into three closely interconnected parts (Figure 1). Below, some of the main considerations related to project design are described, along with activities and the expected outcomes.

### 2.1 Clinical characterization and diagnostics

For practical reasons and to minimize potential confounders, the study includes only one herd. Important considerations were to identify a herd with a diarrhea prevalence and a pathogen load representative for commercial pig production [i.e., 30–50% of the piglets developing diarrhea in the post weaning period, and detectable levels of *Escherichia coli*, *Lawsonia intracellularis* and *Brachyspira pilosicoli* present (Weber et al., 2015)]. Litters from 15 to 18 sows comprising 15 randomly selected piglets are included at six-week intervals (in total litters from 170 sows divided into 11 batches). An important aspect is that antibiotic treatment is closely monitored. Batch treatment with antibiotics is not permitted, and individual treatment is only instigated if diarrhea is accompanied with un-thriftiness. Detailed daily recordings of clinical and anthropometric data are collected electronically for the sows and piglets throughout the project duration. Since intestinal health is the target phenotype of the project, collecting data for monitoring diarrhea is a priority. These data comprise diarrhea-scoring of fecal swabs at birth and subsequently weekly (11 swabs/piglet) and recordings of fecal staining. The data are organized in a database allowing for subsequent epidemiological studies. As an additional layer of characterization, qPCR diagnostics and 16S rRNA gene sequencing will be performed on DNA isolated from the swabs. Data will provide added value by potentially allowing us to establish prognostic microbiota signatures. The data collected provides unprecedented opportunities for identification of different parameters that influence intestinal health. Machine learning methodologies will be used to assess in a holistic manner the robustness of the individual pig with high precision providing distinctively defined phenotypes graduated according to pathogen exposure; extent and cause of diarrhea; and antimicrobial treatment as backbone for the following association studies with the different omics layers.

### 2.2 Genetics and genomics

A biobank has been established which, in addition to the 11 fecal swabs per piglet, will contain fecal samples from the sows collected at farrowing, and from all piglets collected the day before weaning (day 25) and on week 3 after weaning (day 46). At the latter two timepoints blood and serum samples are collected from the piglets for genotyping, metabolomics, transcriptomics, and complete blood cell counts. We will characterize the metabolome, transcriptome and immune-related parameters as well as fecal metagenome sequence data in an integrative manner and across time using samples from the same pigs at the two different time points corresponding to their status before (in most cases) getting diarrhea and during the period where diarrhea is most prevalent. The robustness score and phenotypes, defined based on factors such as the diarrhea-causing pathogen, will serve as the foundation for subsequent association studies with the various metagenomics measured in a spatial intestinal compartment. This approach will enable us to identify key factors influencing intestinal health and robustness.

### 2.3 Pathology and host-pathogen interaction along the intestinal tract

This part of the pillar involves longitudinal compartmentalized sampling of luminal content and tissue along the intestinal tract from a subset of piglets euthanized at different ages (at days 4, 14, 25, 49, and 67). Histopathology, mucus composition, and immune responses will be studied for changes at different ages and in response to disease. Pathological characterization will inform on the clinical observations to further characterize pigs with and without diarrhea. The sampling will enable us to study the interaction between intestinal epithelial cells and the microbiome, allowing identification of key components in both the host and microbiome that are crucial for maintaining homeostasis and understanding host-pathogen networks. The activities within this part of the pillar are integral to the Microbiome Pillar since the intestinal content sampled along the intestinal tract will be used in the Microbiome Pillar for establishing the biogeography of the developing ‘healthy’ and ‘unhealthy’ intestinal microbiome.

## 3 The microbiome pillar

It has long been recognized that the microbiota colonizing the intestine of pigs is an immensely diverse ecosystem, i.e., the intestinal microbiome, that has co-evolved with its host, playing a pivotal role in animal health through its influence on nutritional, physiological and immunological status (16, 17). To this end, research of the past decade has provided a considerable body of information on the spatio-temporal succession of the developing intestinal microbiota in pigs, particularly with respect to the bacterial fraction. However, we have identified important knowledge gaps that hamper our ability to predict and influence intestinal microbiome characteristics underlying robustness and resilience of the developing pig. Whereas knowledge of its bacterial members is well advanced, other members of the microbiota, including archaea, fungi, protozoa and viruses, have until recently received much less attention, mostly for methodological reasons. Only now, particularly owing to technological advances, especially metagenome sequencing based inventories are becoming increasingly available, such as for the pig intestinal virome (18). Having said that, we particularly continue to lack mechanistic understanding of processes driving early assembly and functioning of the intestinal microbiome, as well as the complex molecular interactions within and between cells, and of microorganisms with their host. The Microbiome pillar aims to fill these knowledge gaps by considering the dynamic interactions of the microbiome with the developing pig, as well as the influence of nutritional and environmental factors. The Microbiome pillar is tightly aligned with the other pillars through integrating information from the large prospective phenotyping cohort (Host pillar) with data from small-scale controlled/intensive longitudinal *in vivo* studies (Nutrition pillar) as well as mechanistic *in vitro* and *in vivo* experiments using natural microbiomes, defined microbial consortia, *in vitro* host models as well as a gnotobiotic mouse model, selected luminal metabolites and advanced stem cell based models of the host intestine. State-of-the-art bioinformatics and FAIR (Findable, Accessible, Interoperable, and Reusable) data management will be employed that allow characterization of the antimicrobial resistome as well as to provide strain-level resolution of microbial composition and functionality in complex microbiomes (Data integration pillar).

Research in the microbiome pillar has the following key objectives:

- Establish the biogeography of the developing ‘robust’ and ‘susceptible’ intestinal microbiome during the first 10 weeks of life, with focus on all members, including bacteria, archaea, fungi, protozoa as well as viruses/phages.
- Identify the key members and their functions that govern proper early assembly towards a resilient and robust intestinal ecosystem; establish representative, defined microbiomes of reduced complexity that can be used as models for testing (dietary) interventions and pathogen challenges.
- Establish driving forces of postweaning microbiome resilience and pathogen invasion, particularly in relation to post-weaning diarrhea
- Employ *in vitro* co-culture and *in vivo* gnotobiotic animal models to gain mechanistic understanding of the developing host-microbiome interplay from birth and during the weaning transition, particularly at the mucosal host-microbiome interface.

## 4 The nutrition pillar

Nutrition but also other dietary components including non-nutritional feed additives (such as pre- and probiotics, enzymes) and bioactive substances (such as antioxidants) are the predominant external modulators of the intestinal microbiome and the host and therefore provide a vital opportunity to guide and modulate intestinal health. Progress has been made in our understanding of the multiple purposes of nutrition and dietary components on the host and intestinal microbiome. For instance, a common notion is that feeding a lower protein diet could mitigate PWD in the weaning transition period (19). However, it is similarly noted that such diets do not provide all essential nutrients for supporting an active immune response of the pig, which is necessary to cope with pathogen burden (20). Hence, a major knowledge gap concerns the understanding of how nutrition, including dietary components and derived metabolites, affects the interplay between a healthy intestinal microbiome, epithelial barrier function, and external factors; and how other external physiological stressors contribute to the prevention or development of diarrheal diseases. Therefore, PIG-PARADIGM is investigating the basis for these nutritional and dietary impacts on the gastrointestinal tract to result in new approaches to prevent and mitigate diarrhea risk and result in a robust pig.

The nutrition pillar integrates knowledge on diet and the interactions between dietary components, intestinal tissues, and the gut microbiome, all aimed at strengthening, with piglet gut health and prevention of AMR spread through reduced antibiotic use. Specifically, this pillar encompasses research designed to provide significant, fundamental advances on nutrition-mediated influences on the piglet intestine and gut microbiome. The pillar also incorporates aspects of nutrition and metabolites for the identification of biomarkers to predict and diagnose intestinal inflammation and oxidative distress, which are factors enhancing risk of infection-related diarrhea. This research will deliver innovative dietary interventions and feeding strategies that are relevant under practical conditions, improve and support intestinal health and development, and thereby mitigate the need for antibiotic use. Although sow and piglet nutrition has been extensively studied (21–25), significant gaps remain in knowledge on the diet-intestine-microbiome interface. In this highly integrative, multidisciplinary pillar, we are using hypothesis-driven and exploratory research to investigate the overarching hypothesis that specific diet-mediated intestinal configurations of the microbiome, metabolome, and mucosa predispose piglets to diarrhea, and these configurations can be prevented by nutritional approaches.

This pillar is aligned with the Host pillar to identify and respond to the underlying physiological factors resulting in increased disease risk. This alignment includes the implementation and review of host robustness scores and inclusion of key pathogens identified in that pillar. It is also coordinated with the Microbiome pillar to investigate the causes and consequences of nutrition on microbiome assembly and the use of diet to result in a resilient intestinal microbiome in pigs. Metabolomic and microbiome data generated from the nutrition pillar will be cross-examined in the Data Integration pillar.

Research in the nutrition pillar has the following key focus areas:

1. The impact of diets and nutrients on the small and large intestinal microbiomes and tissues in pre- and post-weaning in pigs.
2. The role of inflammation and oxidative stress on the development of PWD.
3. Modulation of enteric pathogen-permissive intestinal environments towards gut microbiome resilience and pathogen colonization resistance by diet and ingested microorganisms.
4. Dietary strategies that enhance resilience, intestinal digestive capacity and microbiome function during the weaning transition to mitigate host responses which lead to diarrhea.

These focus areas are divided across nine subprojects in four themes that systematically examine individual to holistic components of the diet on PWD through the lens of the gut microbiome. The themes encompass (1) pre-weaning nutrition and piglet gut health; (2) nutrient-microbe-epithelial interactions during the weaning transition; (3) nutrition-based susceptibility to diarrhea in piglets; and (4) diet-based approaches during weaning to improve piglet robustness and diarrhea prevention. Experimental approaches encompass *in vitro* model systems and *in vivo* studies with piglets, including dietary interventions and enterotoxigenic *Escherichia coli* (ETEC) infection. Modulation of the maternal diet will be explored along with investigating dietary supplementation of piglets pre- and post-weaning with a range of dietary components, including iron, prebiotics, amino acids, probiotics and whole foods (e.g., liquid creep feed) in ETEC challenge studies. Studies performed *in vitro* will examine host–microbe interactions mediated through collections of compounds made by members of the gut microbiome (for example, extracellular vesicles and bacteriocins).

## 5 The data integration pillar

The Data Integration pillar will harmonize and integrate the multi-omics host-microbiome-nutrition data generated in the three biological pillars, to holistically model the host-microbiome holobiont in the developing pig. It will generate public data resources for pig gut health researchers. While leveraging the existing scientific domain-specific bioinformatics expertise in the biological pillars and the corresponding research groups generating the datasets, this pillar will also be complemented with deeper (bio)statistical modeling expertise, which will augment the state-of-the-art analysis with novel statistical and machine learning approaches for data integration. It will develop novel algorithms to integrate host-microbiome multi-omics data and derive novel molecular insights that can improve intestinal health of the developing pig. Finally, it will develop resources as well as methods to quantify the AMR gene load (resistome load) in each pig gut metagenome.

### 5.1 Public data resources from PIG-PARADIGM

Data Integration pillar will generate new data resources from PIG-PARADIGM that will help the pig gut health research community. Combining gut metagenome data from PIG-PARADIGM studies with other publicly available data (26), it will create a unified pig gut microbial genome and gene catalogues. These catalogues will include functional annotations and AMR genetic markers, which will help researchers identify and quantify resistome load in metagenomes of interest.

### 5.2 Biomarkers of pig gut health

Using data from numerous studies in the three biological pillars, this pillar will search for accurate biomarkers for the robust pig that resists PWD. Interventions performed under different experimental settings in two continents (dietary interventions in Nutrition Pillar and withdrawal of prophylactic antibiotics in Host Pillar) will help identify global and reproducible biomarkers of robustness. Using a similar approach across studies, the pillar will also develop reliable biomarkers to quantify the resistome load in pig gut microbiomes.

### 5.3 Insights into host-microbial crosstalk in the developing pig

The Data Integration pillar will develop multi-omics data integration approaches to unify and analyze host genetic/expression data, host-microbial metabolomic data and microbiome metagenomic data, to model the host-microbial holobiont. It will develop novel graph-theory-based algorithms (such as graph neural networks) and deep learning algorithms (such as autoencoders) to integrate the multi-omics data and derive novel molecular insights that can improve intestinal health of the developing pig. Our goal is to identify potential interactions between the microbiome, metabolome and mucosa during weaning that predispose piglets to diarrhea, as well as characterize potential remediations. We will integrate metabolome and microbiome profiles with pig gut health metrics, to identify and prioritize features with substantial explanatory power for pig gut health across microbial composition and diet.

### 5.4 Predicting antimicrobial resistome load as an AMR mitigation outcome

The Data Integration Pillar will use resistome load in the pig gut microbiome as a quantitative outcome related to AMR mitigation. The 11-stage execution strategy in the Host Pillar will generate variation in robustness, individualized antibiotic usage, and resistome load between batches. Leveraging this variation, this pillar will model whether increased pig gut health/robustness and decreased use of antibiotics could reduce resistome load. More precisely, it will

- model how robustness and antibiotic usage in an individual pig relates to its own resistome load,
- model how robustness and antibiotic usage of pigs in a pen relate to resistome load in the pen,
- quantify how resistome load will decrease per defined unit of reduction in antibiotic usage.

Additionally, this pillar will investigate the overlap between biomarkers of pig gut resilience from Host Pillar and biomarkers of resistome load to verify whether there is concordance between increased resilience and decrease resistome load. Such concordance may provide statistical evidence that increasing resilience is a logical step to mitigate AMR.

## 6 Conclusion and looking forward

To summarize, across its four pillars, PIG-PARADIGM aims to reduce antibiotic use in pig-production systems - and thereby limit the spread of AMR - by providing the knowledge needed to design and validate dietary and/or microbial therapies that strengthen pig resilience and lower their susceptibility to diarrhea, ultimately reducing the need for antibiotics.

**Figure 2:**
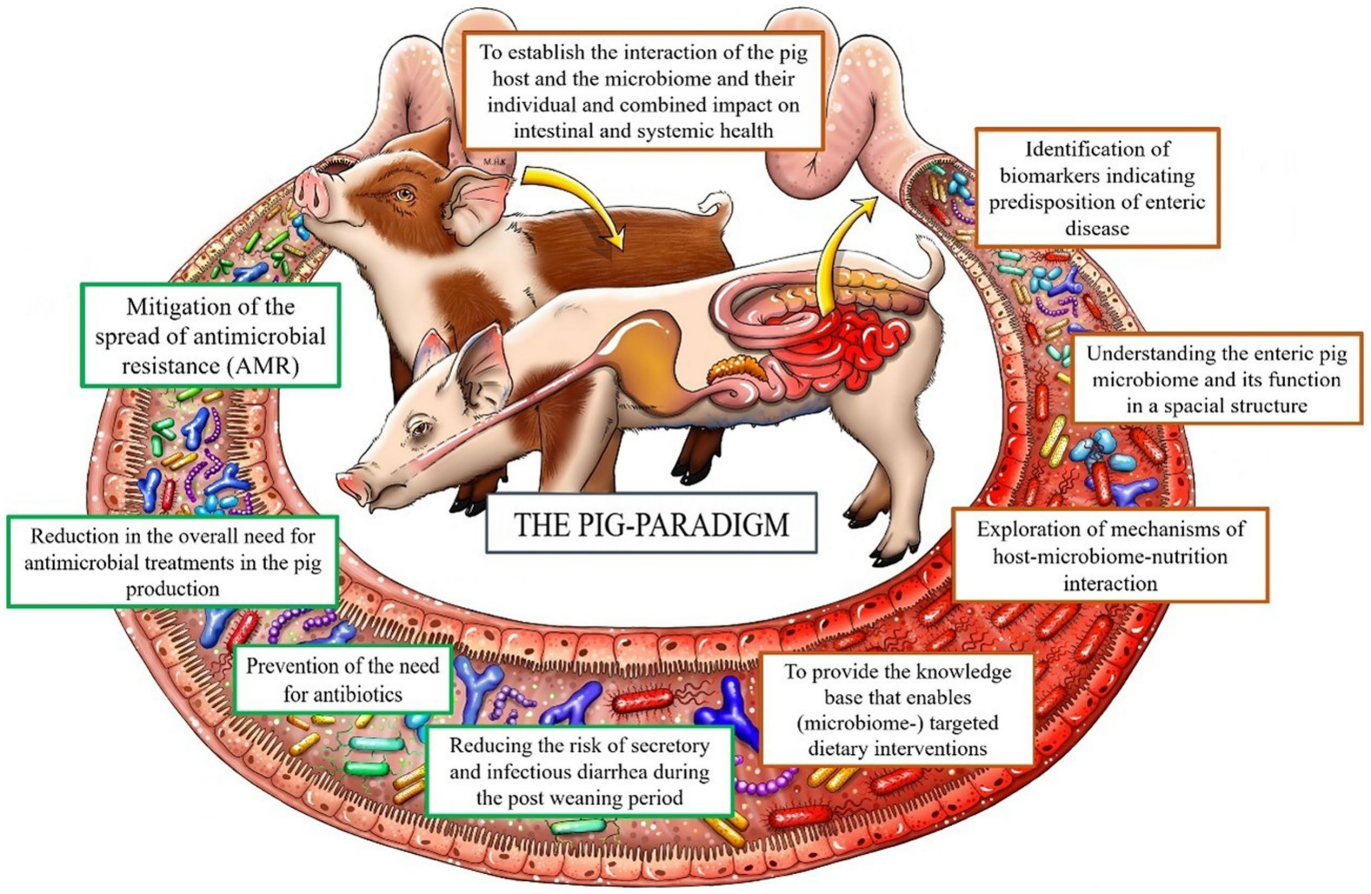
Holistic concept of PIG-PARADIGM visualizing the desired outcomes of the activities as described in the pillars (red boxes) with the desired impact (in green boxes) as summarized above. (Figure created by Maria Kroier).

Hence, PIG-PARADIGM aims to establish an urgently needed fundamental knowledge base of the pig intestinal microbiome and mechanisms underlying its interplay with the growing pig and the diet. We integrate hypothesis-driven and exploratory research to improve our understanding of fundamental driving forces of microbiome development, with the goal to support the development of knowledge-based manipulation of the intestinal microbiome as a powerful tool to maximize resilience of the developing microbiome and its host, and thus minimize the need for antimicrobial therapy, and spread of AMR (Figure 2).

Antimicrobials play an important role in veterinary medicine, just as they do in human medicine. Overuse or misuse of antimicrobials contributes to bacterial resistance, i.e., AMR that causes an ever-increasing threat to global health, food safety, and food security. The animal food production system has been a primary user of antibiotics for growth promotion, and although this was banned in the EU in 2006, conventional pig production, reaching around 25 mio. Pigs/year in Denmark alone, remains a major consumer of antibiotics, and treatment of infections and conventional pig production remains to be a major consumer of antibiotics for the prevention and treatment of infections and therefore is implicated in AMR spread. Antibiotics are the only medicines that can treat bacterial diseases, which means disease prevention is the most effective way to avoid the need for antimicrobials. The overarching strategic aim of PIG-PARADIGM is to reduce the need for antibiotic use and risk of AMR in pork production by deciphering the key determinants of intestinal health in pigs and translating this knowledge into strategies to enhance pig health and robustness and hence avoid the need for antibiotics in the first place. The knowledge obtained from PIG-PARADIGM is expected to be transformative because not only will it result in minimizing antibiotic use and levels of AMR in pig production but will also enable similar approaches to be applied for other food-producing animals or even humans, thereby resulting in further reductions in AMR spread. Further, it is likely that the results of the project will lead to some practical guidelines for the end-users (farmers, veterinarians, industries of the pig farming). However, in both the short and long term there is an urgent need for support of animal health research in relation to the life science research area, as AMR (‘the silent pandemic’) is a global one-health issue that requires collaborative approach of multiple disciplines and bridging of different sectors to counter infectious diseases in both animals and humans.

## Data Availability

The original contributions presented in the study are included in the article/Supplementary material, further inquiries can be directed to the corresponding author.

## Footnotes

1
https://icars-global.org/

## Supplementary material

Supplementary Figure 1 Graphical summary of the historical initiatives to reduce the use of antibiotics in pig production (Figure created by Maria Kroier, Aarhus University).

Supplementary Figure 2 PIG-PARADIGM is organized into Host, Microbiome, and Nutrition pillars, plus a fourth Data Integration pillar that integrates clinical and omics data from the first three biological pillars. More information of the project can be obtained from: https://projects.au.dk/pig-paradigm. (Figure created by Maria Kroier, Aarhus University).
