# Supplementary figures and images for: Reducing antimicrobial resistance burden from livestock production by targeting pig gut health
Source: Front Vet Sci. 2026 Jun 29;13:1854400. doi: 10.3389/fvets.2026.1854400 (PMC13357214; doi:10.3389/fvets.2026.1854400)

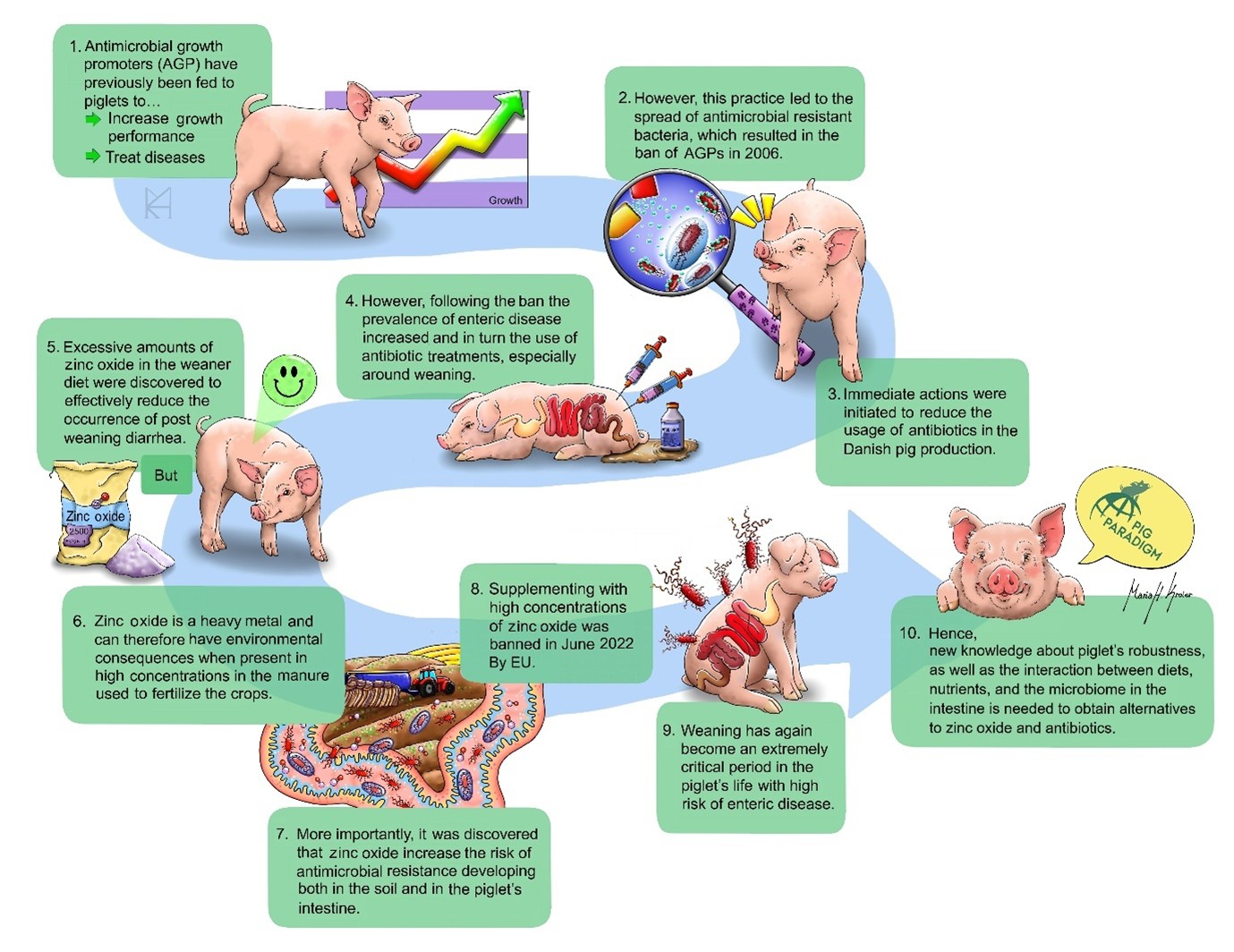

Supplement: Supplementary Figure 1 — Graphical summary of the historical initiatives to reduce the use of antibiotics in pig production (Figure created by Maria Kroier, Aarhus University). [file Image_1.JPEG]

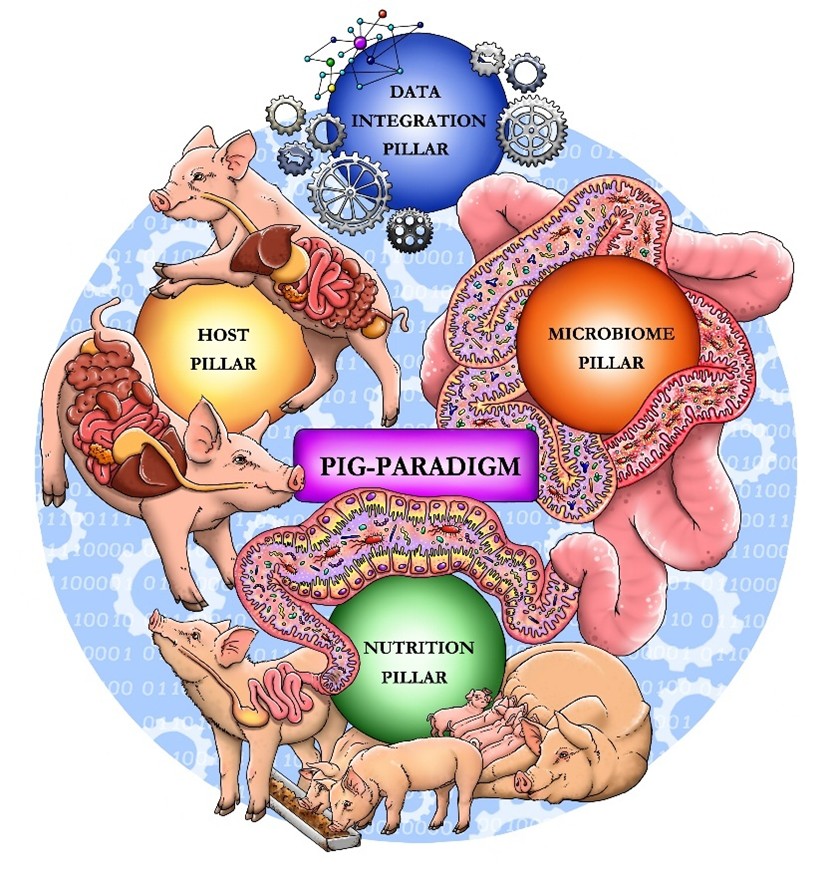

Supplement: Supplementary Figure 2 — PIG-PARADIGM is organized into Host, Microbiome, and Nutrition pillars, plus a fourth Data Integration pillar that integrates clinical and omics data from the first three biological pillars. More information of the project can be obtained from: https://projects.au.dk/pig-paradigm. (Figure created by Maria Kroier, Aarhus University). [file Image_2.JPEG]
